# The complete mitogenome of *Phymorhynchus* sp. (Neogastropoda, Conoidea, Raphitomidae) provides insights into the deep‐sea adaptive evolution of Conoidea

**DOI:** 10.1002/ece3.7582

**Published:** 2021-05-02

**Authors:** Mei Yang, Dong Dong, Xinzheng Li

**Affiliations:** ^1^ Institute of Oceanology Chinese Academy of Sciences Qingdao China; ^2^ Center for Ocean Mega‐Science Chinese Academy of Sciences Qingdao China; ^3^ University of Chinese Academy of Sciences Beijing China; ^4^ Laboratory for Marine Biology and Biotechnology Qingdao National Laboratory for Marine Science and Technology Qingdao China

**Keywords:** adaptive evolution, Conoidea, deep‐sea, mitogenome, *Phymorhynchus*

## Abstract

The deep‐sea environment is characterized by darkness, hypoxia, and high hydrostatic pressure. Mitochondria play a vital role in energy metabolism; thus, they may endure the selection process during the adaptive evolution of deep‐sea organisms. In the present study, the mitogenome of *Phymorhynchus* sp. from the Haima methane seep was completely assembled and characterized. This mitogenome is 16,681 bp in length and contains 13 protein‐coding genes, 2 rRNAs, and 22 tRNAs. The gene order and orientation were identical to those of most sequenced conoidean gastropods. Some special elements, such as tandem repeat sequences and AT‐rich sequences, which are involved in the regulation of the replication and transcription of the mitogenome, were observed in the control region. Phylogenetic analysis revealed that Conoidea is divided into two separate clades with high nodal support. Positive selection analysis revealed evidence of adaptive changes in the mitogenomes of deep‐sea conoidean gastropods. Eight residues located in *atp6*, *cox1*, *cytb*, *nad1*, *nad4*, and *nad5* were determined to have undergone positive selection. This study explores the adaptive evolution of deep‐sea conoidean gastropods and provides valuable clues at the mitochondrial level regarding the exceptional adaptive ability of organisms in deep‐sea environments.

## Graphical abstract

In this paper, the mitogenome of Phymorhynchus sp. from the Haima methane seep was completely assembled and characterized. We also discussed the phylogenetic relationships and gene arrangements of the superfamily Conoidea. Furthermore, eight residues located in atp6, cox1, cytb, nad1, nad4, and nad5 genes were inferred to be positively selected sites along the branches leading to deep‐sea conoidean gastropods, which indicate that the related genes were potentially under positive selection pressure.

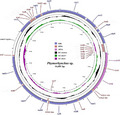

## 1 INTRODUCTION

Conoidea is the most diverse Neogastropoda superfamily (Bouchet et al., 2017; Uribe et al., 2018) and harbors 17 extant recognized families, nearly 400 accepted genera and more than 5,000 existing species (WoRMS, http://www.marinespecies.org/). Conoidean gastropods are characterized by the presence of a venom gland and specialized radular system, which facilitates prey capture and deters predators (Dutertre et al., 2014; Fujikura et al., 2009). These evolutionary features were speculated to provide a great advantage for colonization by these species of the marine realm from polar regions to tropical areas and from shallow coastal waters to the deep sea (Kantor et al., 2016; Uribe et al., 2017; Warén & Bouchet, 2009).

Due to the abundant species and the extensive homoplasy among shell and anatomical characters, the classification of Conoidea has remained problematic, and relevant studies have been limited mainly by the absence of a consistent phylogenetic hypothesis. More recently, based on extensive sampling, progress has been made in the Conoidea taxonomy and phylogeny (Abdelkrim et al., 2018; Bouchet et al., 2011; Puillandre et al., 2008; Uribe et al., 2018). One of the outcomes was that the original family Turridae (sensu) (Powell, 1966) was separated into 13 monophyletic families, the largest of which was Raphitomidae, which included 73 genera (WoRMS). Among those genera, the most well‐known taxon in cold seep and hydrothermal vent environments is the genus *Phymorhynchus*. To date, 19 species of *Phymorhynchus* have been recorded in the WoRMS, and approximately 12 species of *Phymorhynchus* from cold seeps and hydrothermal vents have been described (Zhang & Zhang, 2017).

The mitogenome is characterized by conserved gene content, useful evolutionary information, rare recombination events, and a relatively high evolutionary rate (Barr et al., 2010; Boore, 1999; Hao et al., 2010), and it has been established as a useful tool in studies of genetic diversity, phylogeny, molecular evolution, and phylogeography at various taxonomic levels (Gissi et al., 2008; Lee et al., 2019; Shen, Kou, et al., 2017; Shen, Wei, et al., 2017). Recently, several studies have demonstrated the unique evolutionary process and adaptations of mitogenomes from organisms in extremely harsh habitats, such as the ATP synthase genes of Tibetan loaches and Mariana Trench starfish (Mu et al., 2018a; Wang, Shen, et al., 2016), the cytochrome *b* gene of high‐altitude alpaca and deep‐sea vesicomyid bivalves (da Fonseca et al., 2008; Yang et al., 2019), the cytochrome *c* oxidase genes of wild yak and Tibetan antelope (Wang, Shen, et al., 2019; Wang, Tang, et al., 2019; Xu et al., 2005), and the NADH dehydrogenase genes of Chinese snub‐nosed monkeys and deep‐sea crab (Yu et al., 2011; Zhang, Wu, et al., 2020; Zhang, Gao, et al., 2020).

The deep sea represents the most extensive ecosystem on Earth; however, it is a harsh environment characterized by a lack of sunlight and hypoxic, high hydrostatic pressure, and low‐temperature conditions (Rex, 1981; Sanders & Hessler, 1969). The organisms living in these areas generally display a series of physiological and biochemical adaptations, the molecular mechanisms of which have attracted the interest of scientists, such as chemosynthetic symbioses in bivalves, adaptive visual metamorphosis in deep‐sea hydrothermal vent crabs, the expansion of gene families in deep‐sea mussels, and a series of putatively selected codons in the mitochondrial protein‐coding genes of Bythograeidae crabs (Duperron et al., 2013; Jinks et al., 2002; Sun et al., 2017; Wang, Shen, et al., 2019; Wang, Tang, et al., 2019). Aerobic respiration is the basis of all organismal activity, and several components of oxidative phosphorylation (OXPHOS) are encoded by mitochondrial genes (Boore, 1999). Hypoxia is a major threat to the OXPHOS pathway, which could result in cell death. Therefore, the extreme deep‐sea environment has the potential to affect the mitochondrial genome (mitogenome). It is necessary and important to investigate unusual mitogenomic features and mitochondrial protein‐coding genes (PCGs) for energy metabolism to understand the adaptive molecular mechanisms of organisms living in deep‐sea environments.

With the development of high‐throughput sequencing, Conoidea mitogenome information has been continuously accumulated in public databases. In the present study, we report the complete mitogenome of one conoidean gastropod, *Phymorhynchus* sp., which was collected from the Haima cold seep in the South China Sea. We aimed to (a) characterize the mitogenome organization, features, codon usage, and gene arrangement of *Phymorhynchus* sp.; (b) explore the phylogenetic relationships of conoidean gastropods based on 13 mitochondrial protein‐coding gene sequences; and (c) evaluate the selective pressure operating on mitochondrial PCGs of deep‐sea conoidean gastropods to understand the genetic basis and adaptive evolutionary architecture of deep‐sea organisms.

## 2 MATERIALS AND METHODS

### 2.1 Sampling and DNA extraction

The *Phymorhynchus* sp. was collected from the Haima methane seep on the northwestern slope of the South China Sea at a depth of 1,380 m using a remotely operated vehicle (ROV) in May 2018. The specimen was frozen in liquid nitrogen and then preserved at −80°C until DNA extraction. Total genomic DNA was extracted from muscle tissues using an E.Z.N.A. Tissue DNA Kit (OMEGA, China) following the manufacturer's instructions.

### 2.2 Mitogenome sequencing, assembly, and annotation

A paired‐end library with an insert size of 450 bp was generated from total genomic DNA using a TruSeq™ Nano DNA Sample Prep Kit (Illumina) and then sequenced on the Illumina HiSeq 4000 platform (2 × 150 bp paired‐end reads).

Quality control of the raw data was performed using Trimmomatic (Bolger et al., 2014) by removing adapters, duplicated sequences, reads with a quality score below 20 (Q < 20), and reads containing a percentage of uncalled bases (“N” characters) equal to or greater than 10%. The clean data were assembled into contigs using SPAdes (Bankevich et al., 2012) with default parameters. After that, we blasted the contigs against the reference mitogenomes from species of Conoidea shown in Table S1. The contigs identified as mitogenome sequences were manually examined for repeats, and then, circular mitochondrial DNA was established.

The assembled mitogenome was preliminarily annotated using the MITOS web server (http://mitos.bioinf.uni‐leipzig.de/index.py) (Bernt et al., 2013). The protein‐coding genes were analyzed with ORF finder (http://www.ncbi.nlm.nih.gov/gorf/gorf.html), which is available from the NCBI, using the invertebrate mitochondrial genetic code, and the exact initiation and termination codon positions were manually determined accordingly. The boundaries of the ribosomal RNA (rRNA) genes were confirmed by alignment with homologous genes of conoidean gastropods. Transfer (tRNA) genes and their secondary structures were predicted by ARWEN (Laslett & Canbäck, 2008) and tRNAscan (Lowe & Eddy, 1997). The organellar genome DRAW (Lohse et al., 2007) was used to create a circular display of the *Phymorhynchus* sp. mitogenome.

### 2.3 Sequence analysis

The nucleotide composition was determined using DnaSP (Librado & Rozas, 2009). The AT and GC skew values were calculated with the following formulas: AT skew = (A − T)/(A + T) and GC skew = (G − C)/(G + C) (Perna & Kocher, 1995), with A, T, G, and C representing the contents of the four bases. The frequencies of both codons and amino acids and relative synonymous codon usage (RSCU) values were measured using MEGA 6 (Tamura et al., 2013). Tandem Repeats Finder (http://tandem.bu.edu/trf/trf.html) (Benson, 1999) was employed to search for the tandem repeat sequences in the long noncoding regions, and their potential secondary structures were inferred by the Mfold web server with default parameters (http://unafold.rna.albany.edu/?q=mfold) (Zuker, 2003).

### 2.4 Phylogenetic analyses

Phylogenetic analyses of Conoidea were performed based on the mitogenomes of *Phymorhynchus* sp. and thirty‐one other conoidean gastropods belonging to fourteen distinct families available in GenBank (Table S1). *Nassarius javanus*, *Tritia reticulata,* and *Bolinus brandaris* from Buccinoidea and Muricoidea served as outgroups. The nucleotide sequences of 13 mitochondrial PCGs from the aforementioned mitogenomes were aligned in batches with MAFFT (Katoh & Standley, 2013) using the codon alignment mode. Ambiguously aligned regions were removed using Gblocks 0.91b (Talavera & Castresana, 2007) with the default settings. The resulting alignments were concatenated into a single dataset with PhyloSuite (Zhang, Wu, et al., 2020; Zhang, Gao, et al., 2020). The best partition schemes and optimal substitution models were selected by PartitionFinder 2 (Lanfear et al., 2016) with the greedy algorithm and corrected Akaike information criteria (AICc). The best substitution models applied to each partition are listed in Table S2. Phylogenetic relationships were determined using both the maximum‐likelihood (ML) and Bayesian inference (BI) methods. The ML phylogenies were inferred using IQ‐TREE (Nguyen et al., 2015) with the models selected for each partition (including FreeRate models), and the branch supports were assessed by ultrafast bootstrap with 1,000 replicates (Hoang et al., 2018). The Bayesian tree was constructed using MrBayes 3.2.6 (Ronquist et al., 2012). Two independent runs were carried out with four Markov chain Monte Carlo (MCMC) chains for 10^6^ generations, with sampling every 100 generations. The average standard deviation of split frequencies and the likelihood values were monitored, to determine whether the two runs converged onto the stationary distribution. The initial 25% of the trees generated prior to the achievement of stationarity of the log‐likelihood values were discarded as burn‐in. The remaining trees were used to estimate the 50% majority rule consensus tree and the Bayesian posterior probabilities (PPs). The effective sample size (ESS) values for all parameters were checked with Tracer 1.7 (http://www.beast2.org/treeannotator/) to ensure convergence. The phylograms and gene orders were graphically edited with iTOL (Letunic & Bork, 2007).

### 2.5 Adaptive evolution analysis

Estimating the nonsynonymous/synonymous substitution ratios (ω = *dN*/*dS*) is considered a useful approach for quantifying the impact of natural selection on adaptive evolution (Ohta, 1992). The values of ω indicate changes in selection pressure, where ω >1, =1, and <1 correspond to positive selection, neutrality, and purifying selection, respectively (Yang, 1998). In this study, the codon‐based maximum‐likelihood (CodeML) method implemented in PAML (Yang, 2007) was applied to determine whether adaptive evolution might have occurred in the mitochondrial PCGs of deep‐sea conoidean gastropods (Table S3).

We first used branch models to evaluate the selective pressure among the examined gastropoda lineages. The one‐ratio model (M_0_), which was used as the null hypothesis, assumes the same ω for all the branches. The free‐ratio model (M_1_) allows each branch to have a different ω, and the two‐ratio model (M_2_) assumes that the branches of interest (i.e., the foreground lineages) have different ω values than the background lineages. Here, M_1_ and M_0_ were compared to test whether different lineages in the tree had different ω values, and M_2_ was compared with M_0_ to determine whether deep‐sea conoidean gastropods were subjected to more selection pressure than other gastropods in shallow water. In M_2_, ω_0_ and ω_1_ represent the background lineage values of the shallow water gastropods in the phylogeny and deep‐sea conoidean gastropods, respectively. To test whether M_1_ or M_2_ fit the data significantly better than M_0_, pairwise models were compared using critical values of the chi‐square (χ^2^) distribution and likelihood ratio tests (LRTs). The test statistic was estimated as twice the difference in log likelihood (2ΔL), and the degrees of freedom were estimated as the difference in the number of parameters for each model.

Furthermore, since positive selection often occurs over a short period of evolutionary time and/or at a few sites, branch‐site models were introduced to investigate positive selection along the prespecified lineages (Zhang et al., 2005). Branch‐site model A (positive selection model) was used to identify the positively selected sites among the deep‐sea conoidean gastropods (marked as the foreground lineage). The presence of sites with ω > 1 suggests that model A fits the data significantly better than the corresponding null model A. The Bayes empirical Bayes (BEB) approach was used to calculate posterior probabilities and identify amino acid sites under positive selection following significant LRTs (Yang et al., 2005).

Finally, TreeSAAP (Woolley et al., 2003), which compares nonsynonymous residue property changes and identifies positive selection amino acid properties, was used to validate the results of CodeML at the protein physicochemical level. Changes were detected by TreeSAAP based on 31 physicochemical amino acid properties, and all of the changes identified had a magnitude of 6–8. In addition, *p* values ≤0.01 were used as an index for measuring the degree of radical amino acid substitution.

### 2.6 Mapping positive selection sites onto protein structures

To provide further insights into the functional significance of the putative positive selection sites, we mapped them onto secondary and three‐dimensional protein structures. Raptor X (http://raptorx.uchicago.edu/) (Källberg et al., 2014) was applied to predict the secondary structures of the related proteins. The PredictProtein web server (https://ppopen.rostlab.org/) (Yachdav et al., 2014) was used to predict the protein domains. I‐TASSER (Zhang, 2008) was employed to predict the 3D structures of related proteins under positive selection. Finally, the putative positive selection sites were mapped onto the corresponding 3D structures using PyMOL.

## 3 RESULTS AND DISCUSSION

### 3.1 Mitogenome organization

**FIGURE 1:**
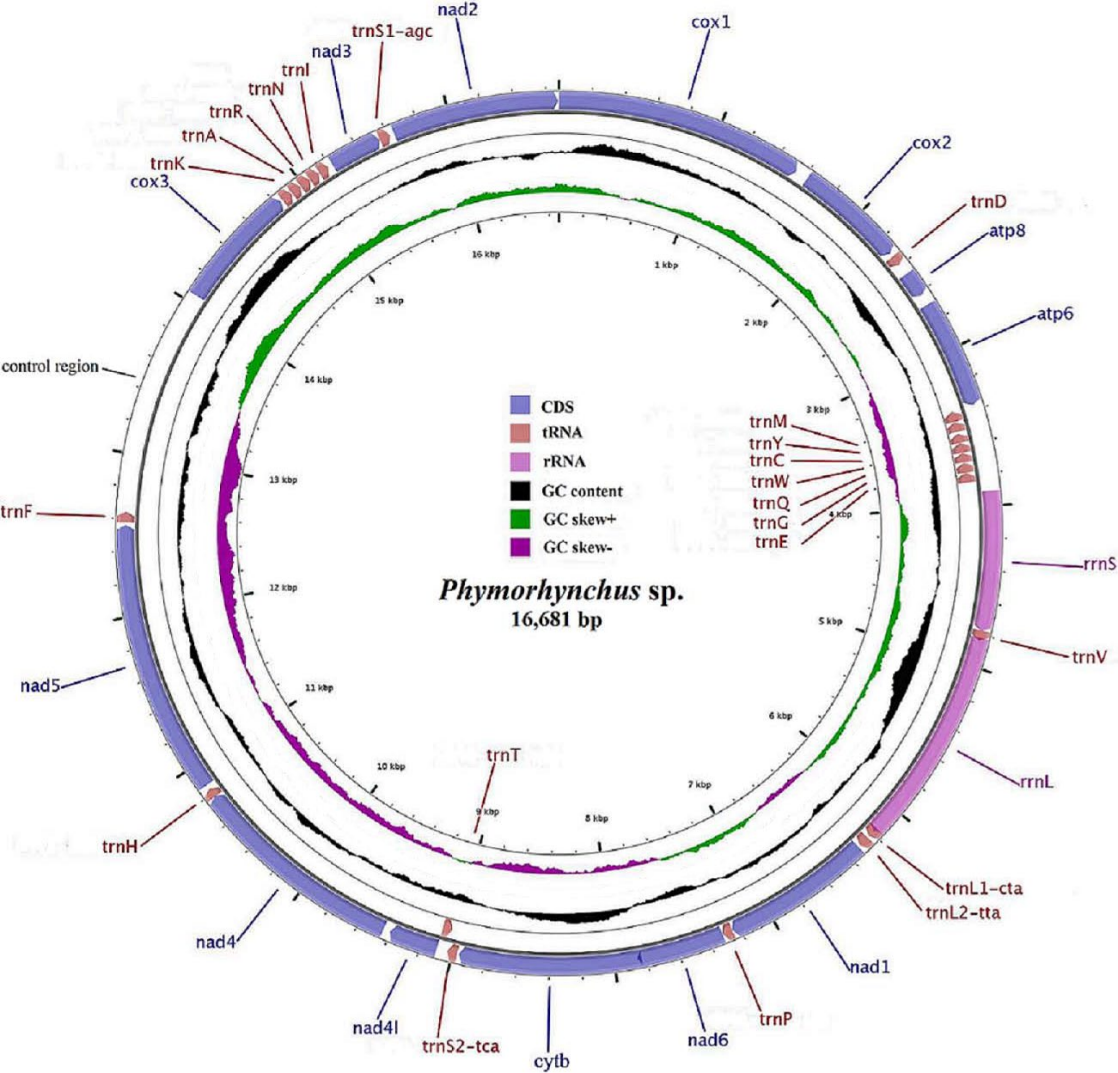
The organization of the mitogenome of Phymorhynchus sp. Genes for proteins and rRNAs are shown with standard abbreviations. Genes for tRNAs are represented by a single letter for the corresponding amino acid, with two leucine tRNAs and two serine tRNAs differentiated by numerals. Genes encoded by the H strand were showed outside the circle, and those encoded by the L strand were showed inside the circle. The inner ring and the middle ring, respectively, showed the GC skew and GC content in the mitogenome

A total of 32,149,089 clean reads were generated by Illumina HiSeq sequencing with an insert size of approximately 450 bp. After assembly, we obtained the complete mitogenome of *Phymorhynchus* sp., with a length of 16,681 bp (GenBank accession number: MN840973). The mitogenome is a circular molecule containing 37 genes, including 13 PCGs, 2 rRNA genes, and 22 tRNA genes. Among these genes, 8 of the 22 tRNAs (*trnM*, *trnY*, *trnC*, *trnW*, *trnQ*, *trnG*, *trnE,* and *trnT*) were encoded by the light (L) strand, while the remaining genes were encoded by the heavy (H) strand (Figure 1; Table 1). Furthermore, 7 overlaps between adjacent genes were detected, with sizes ranging from 2 to 26 bp (Table 1).

**TABLE 1:** Mitogenome organization of *Phymorhynchus* sp

Name	Strand	Range	Size		Codon			Intergenic nucleotides^a^ (bp)
			Nucleotides	Amino acid	Start	Stop	Anticodon	
*cox1*	H	1–1,539	1,539	512	ATG	TAG		–
*cox2*	H	1,618–2,304	687	228	ATG	TAA		78
*trnD*	H	2,329–2,395	67				GTC	24
*atp8*	H	2,465–2,626	162	53	ATG	TAG		69
*atp6*	H	2,693–3,370	678	225	ATG	TAA		66
*trnM*	L	3,373–3,438	66				CAT	2
*trnY*	L	3,441–3,506	66				GTA	2
*trnC*	L	3,519–3,580	62				GCA	12
*trnW*	L	3,581–3,645	65				TCA	0
*trnQ*	L	3,647–3,709	63				TTG	1
*trnG*	L	3,712–3,776	65				TCC	2
*trnE*	L	3,777–3,841	65				TTC	0
*rrnS*	H	3,909–4,775	867					67
*trnV*	H	4,773–4,836	64				TAC	−3
*rrnL*	H	4,818–6,185	1,368					−19
*trnL1* ^a^	H	6,161–6,229	69				TAG	−25
*trnL2* ^a^	H	6,241–6,308	68				TAA	11
*nad1*	H	6,321–7,250	930	309	ATC	TAA		12
*trnP*	H	7,251–7,314	64				TGG	−2
*nad6*	H	7,316–7,858	543	180	ATG	TAA		−3
*cytb*	H	7,833–8,960	1,128	375	ATT	TAA		−26
*trnS2* ^a^	H	8,971–9,034	64				TGA	10
*trnT*	L	9,035–9,099	64				TGT	0
*nad4l*	H	9,100–9,405	306	101	ATA	TAG		0
*nad4*	H	9,447–10,778	1,332	443	ATG	TAA		41
*trnH*	H	10,774–10,834	61				GTG	−5
*nad5*	H	10,862–12,562	1,701	566	ATC	TAG		27
*trnF*	H	12,578–12,645	68				GAA	15
*contral region*	H	12,646–14,054	1,409					0
*cox3*	H	14,055–14,834	780	259	ATG	TAA		0
*trnK*	H	14,846–14,913	68				TTT	11
*trnA*	H	14,918–14,983	66				TGC	4
*trnR*	H	14,986–15,053	68				TCG	2
*trnN*	H	15,056–15,121	66				GTT	2
*trnI*	H	15,127–15,192	66				GAT	5
*nad3*	H	15,222–15,548	327	108	ATT	TAG		29
*trnS1* ^a^	H	15,550–15,617	68				GCT	1
*nad2*	H	15,645–16,676	1,032	343	ATG	TAA		27

^a^ Intergenic nucleotides refer to noncoding bases between two adjacent genes, and a negative value indicates an overlap.

The base composition of the *Phymorhynchus* sp. mitogenome is as follows: A, 31.20%; C, 14.72%; G, 15.76%; and T, 38.32%. The AT content (69.52%) was distinctly higher than the GC content (30.48%); this AT richness is typical in many other conoidean species (Table S4). A negative AT skew (−0.102) and a positive GC skew (0.034) were observed in the *Phymorhynchus* sp. mitogenome, which is opposite to the trends found in most other conoidean species and indicates bias toward T and G (Table S4).

### 3.2 Protein‐coding genes

The mitogenome of *Phymorhynchus* sp. contains 13 PCGs with a total length of 11,145 bp that encode 3,702 amino acids, and the AT content was 67.9% (Table S4). Similar to most metazoan mitogenomes, all of the PCGs in *Phymorhynchus* sp. were initiated by typical ATN codons (8 with ATG, 2 with ATC, 2 with ATT, and 1 with ATA) and ended by complete TAA or TAG termination codons (Table 1) (Wolstenholme, 1992). However, incomplete stop codons (T/TA) are frequently detected in other conoidean gastropod mitochondrial genes (Uribe et al., 2017; Uribe et al., 2018) and presumed to be corrected by posttranscriptional polyadenylation (Dreyer & Steiner, 2006; Ojala et al., 1981).

**FIGURE 2:**
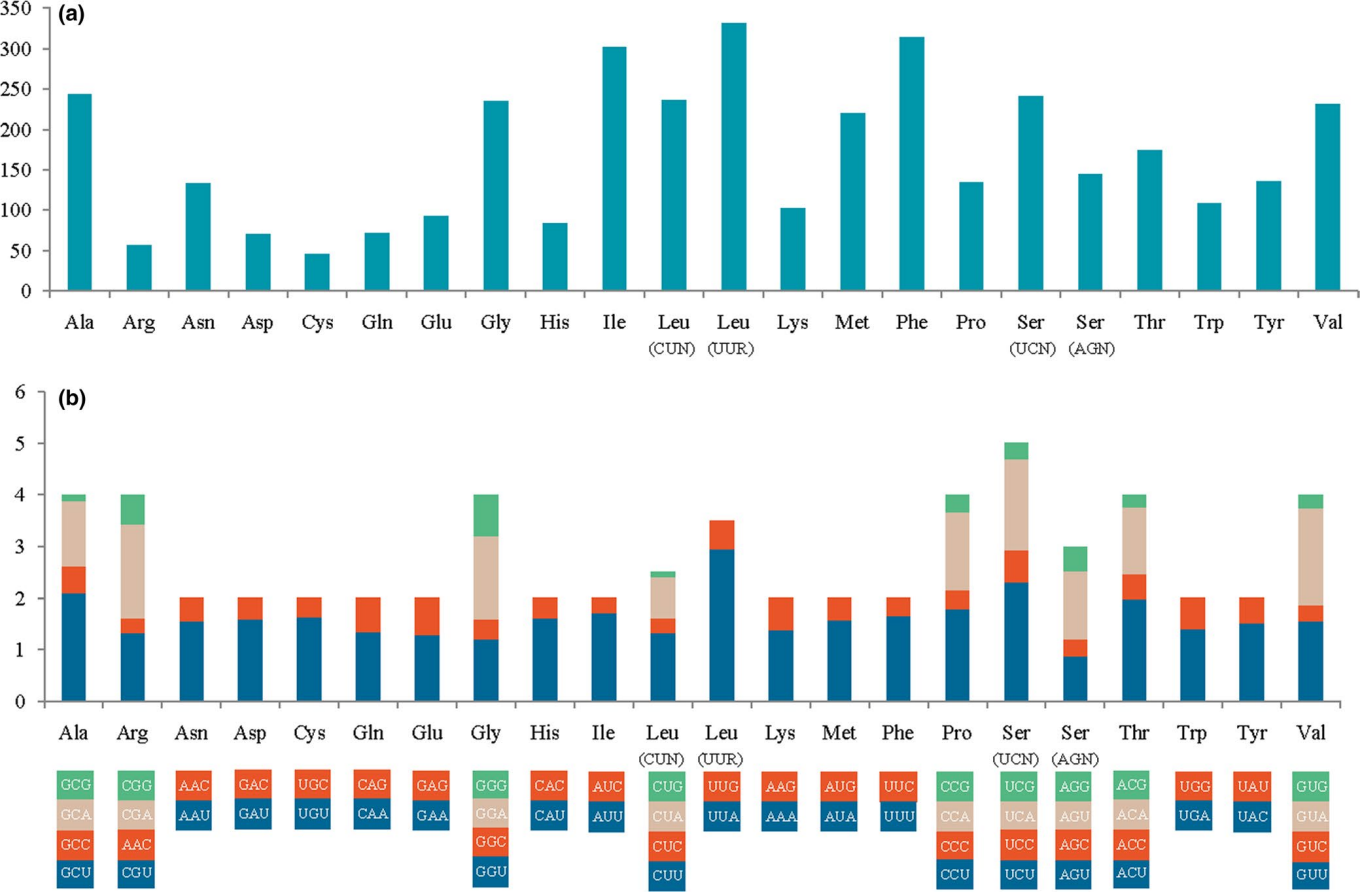
Codon usage (A) and RSCU (B) of the *Phymorhynchus* sp. mitogenome. Numbers to the left refer to the total number of codons (A) and the RSCU values (B). Codon families are plotted on the x‐axis

The amino acid usage and RSCU values in the PCGs of *Phymorhynchus* sp. are summarized in Figure 2. Large numbers of studies have shown that metazoan mitogenomes usually have a bias toward a higher representation of nucleotides A and T, which leads to a subsequent bias in the corresponding encoded amino acids (Salvato et al., 2008; Wang, Chao, et al., 2016; Yu & Li, 2012). Similarly, in the *Phymorhynchus* sp. PCGs, the amino acids encoded by A + T‐rich codon families (Asn, Ile, Lys, Met, Phe, and Tyr) have a higher frequency of use than those encoded by G + C‐rich codon families (Ala, Arg, Gly, and Pro) (Figure 2). Because the tRNAs were duplicated, Leu (15.3%) and Ser (10.4%) were the most frequently used, accounting for more than a quarter of the total PCGs, while Arg (1.5%) and Cys (1.2%) were the least frequently used, accounting for <3% of the total PCGs. The RSCU values indicate that the five most commonly used codons are TTA (Leu), TCT (Ser), GCT (Ala), ACT (Thr), and GTA (Val) (Figure 2), which shows the A + T bias at the third codon position. This result supports the hypothesis that codons with A and T in the third position are used more frequently than other synonymous codons in metazoan mitogenomes (Salvato et al., 2008; Wang, Chao, et al., 2016; Yang et al., 2019).

### 3.3 Ribosomal and transfer RNA genes

The *rrnS* and *rrnL* genes of *Phymorhynchus* sp. are 867 bp (AT% = 70.9) and 1,368 bp (AT% = 74.3) in length, respectively. *rrnS* is located between *trnE* and *trnV*, while *rrnL* is located between *trnV* and *trnL1^cta^*, and this pattern (*rrnS*‐*trnV*‐*rrnL*) has been found in most conoidean mitogenomes (Uribe et al., 2018).

The classical set of 22 tRNA genes was identified in the mitogenome of *Phymorhynchus* sp., with lengths ranging from 62 (*trnC*) to 69 bp (*trnL1^cta^*) (Table 1). The AT content of the tRNA genes was 70.4% (Table S4). The secondary structures of the tRNA genes, all of which could be folded into typical clover‐leaf structures, are schematized in Figure S1.

### 3.4 Noncoding regions

A total of 1,929 bp of noncoding nucleotides that vary in length from 1 to 1,409 bp were scattered among 25 intergenic regions (Table 1). The longest intergenic sequence (1,409 bp) is located between the *trnF* and *cox3* genes; this sequence was identified as the putative control region (CR) and has an A + T content of 72.1%.

**FIGURE 3:**
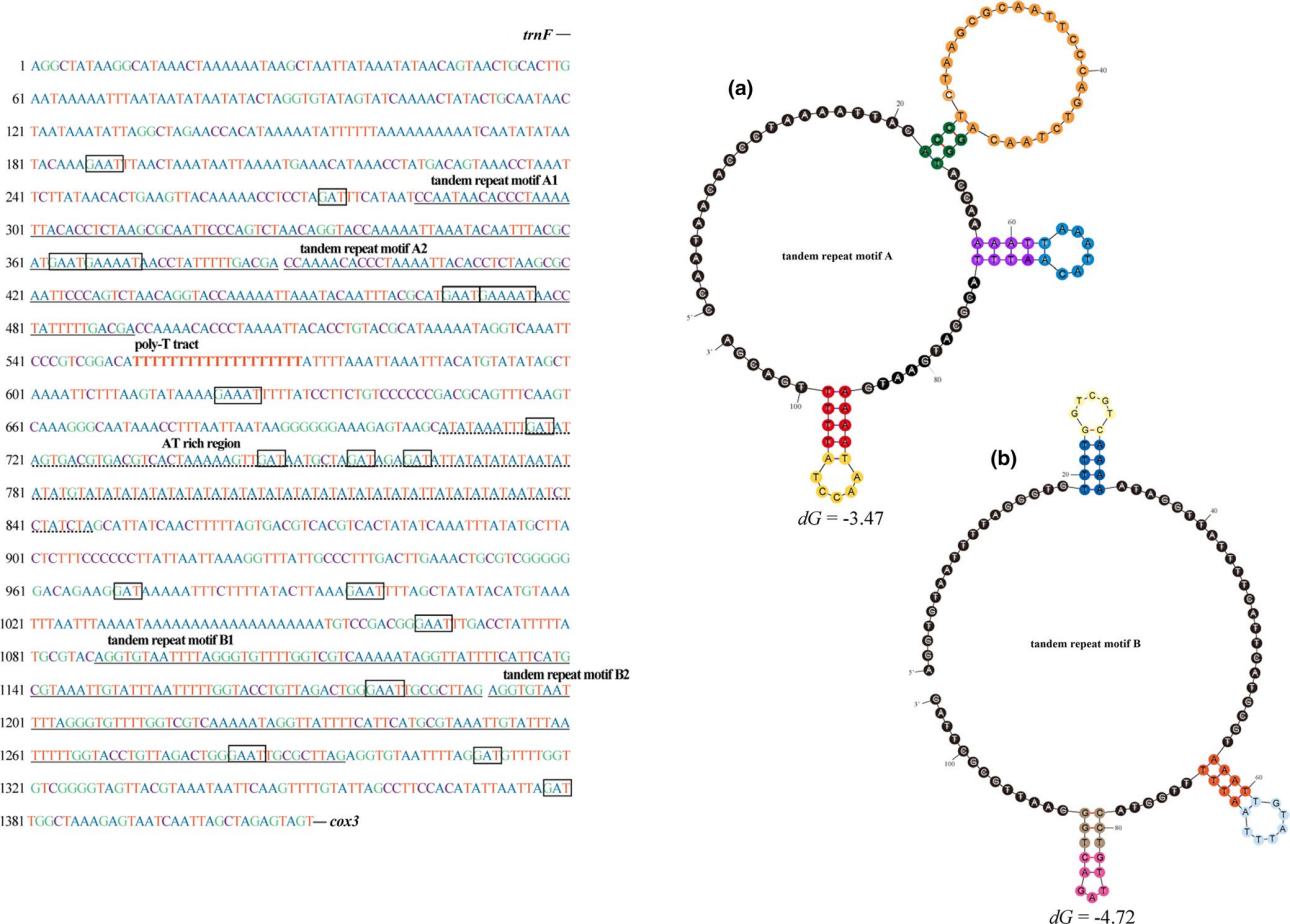
Nucleotide sequences and stem‐loop structures of the tandem repeat motifs (A and B) in the control region (CR) of the *Phymorhynchus* sp. mitogenome. The CR is flanked by sequences encoding *trnF* and *cox3*. The CR consists of certain patterns, such as special G(A)nT motifs (marked with a box), poly‐T tract, and AT‐rich regions (marked with dotted line)

The remarkable feature of the control region for *Phymorhynchus* sp. is the existence of tandem repeat units (Figure 3). Tandem repeat motif A is 209 bp in length (positions 12,929–13,137), comprising two distinct tandem repeat units 105 bp (tandem repeat motif A1) to 104 bp (tandem repeat motif A2), while tandem repeat motif B is 208 bp in length (positions 13,730–13,937) and consists of two identical tandem repeat units of 104 bp (tandem repeat motifs B1 and B2) (Figure 3). These tandem repeat sequences could be folded into stem‐loop secondary structures with minimal free energy (Figure 3) and are thought to function as promoters and transcriptional regulators (Fernández‐Silva et al., 2003; Flot & Tillier, 2007; Stanton et al., 1994). In addition, conserved elements of typical control regions, such as “G(A)_n_T” motifs and AT‐rich sequences, were also identified in *Phymorhynchus* sp. mitogenome.

### 3.5 Phylogenetic relationships and gene arrangements

**FIGURE 4:**
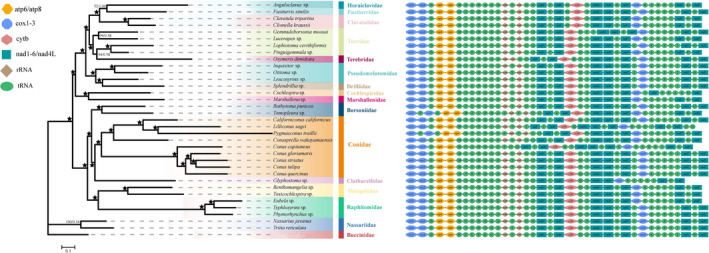
Phylogenetic tree inferred from the partitioned nucleotide sequences of 13 mitochondrial PCGs based on the Bayesian and maximum‐likelihood methods. Nodal supports are denoted on the corresponding branches, and the black asterisk (★) indicates both posterior possibilities and ultrafast bootstrap values ≥95% for the node. The gene orders of mitogenomes of the studied species mapped onto the phylogenetic tree. Genes encoded by the light strand are prefixed with minus signs

The phylogenetic analyses of conoidean gastropods based on 13 concatenated mitochondrial protein‐coding gene sequences using ML and BI resulted in almost identical topologies with varying levels of support (Figure 4). The monophyly of Conoidea was previously confirmed by the phylogenetics of three mitochondrial genes with moderate‐to‐high support values (Puillandre et al., 2008, 2011). Here, the superfamily Conoidea is divided into two separate clades with high nodal support (bootstrap values >95 and posterior possibilities = 1), which are termed “Clade I” and “Clade II.” Clade I includes the families Clavatulidae, Cochlespiridae, Drilliidae, Fusiturridae, Horaiclavidae, Marshallenidae, Pseudomelatomidae, Terebridae, and Turridae. Furthermore, Cochlespiridae and Marshallenidae are grouped together with high support and are sister to the rest of Clade I, which is composed of three main lineages: (a) Clavatulidae, Fusiturridae, and Horaiclavidae; (b) Terebridae and Turridae; and (c) Drilliidae and Pseudomelatomidae (Figure 4).

Clade II included the families Borsoniidae, Clathurellidae, Conidae, Mangeliidae, and Raphitomidae. The phylogenetic analyses showed that (a) *Phymorhynchus* sp. is clustered with *Eubela* sp. and *Typhlosyrinx* sp. in the Raphitomidae; and (b) *Glyphostoma* sp. is the only representative of Clathurellidae and has a monophyletic relationship with Conidae, which is the sister group to Borsoniidae (Figure 4).

In view of the extraordinary taxonomic and ecological diversity of conoidean gastropods, phylogenetic analyses have tended to focus on a single family (Abalde et al., 2019; Fu et al., 2019; Uribe et al., 2017); thus, a comprehensive and statistically robust phylogenetic framework of the superfamily Conoidea has not been previously performed. Based on 34 complete or nearly complete mitogenomes, Uribe et al. (2018) first explored the relationships within Conoidea at the family level and recovered two main monophyletic groups with strong support. Then, based on the exon capture phylogeny, Abdelkrim et al. (2018) revealed new relationships among major groups of Conoidea, which contradicted the previously published trees of Uribe et al. (2018) in a few cases. For example, in a tree based on an exon capture strategy, Cochlespiridae is recognized for the first time as the sister group of all other Conoidea and not the sister group of the other members of clade A (Uribe et al., 2018). Generally, different molecular markers and sampling coverage can impact the inferred phylogenetic relationships. In the existing phylogenetic analysis, several families are represented by only one or two species or genera each; therefore, more comprehensive taxon samplings and more genetic data will be necessary to reach the goal of reconstructing the natural evolutionary history of Conoidea.

The gene order found in the mitogenome of *Phymorhynchus* sp. conforms to the consensus genome organization for Neogastropoda (Figure 4) (Cunha et al., 2009). Generally, gastropod mitogenomes exhibit a great variety of gene orders compared with other metazoan mitogenomes; nevertheless, for the majority of species within a main gastropod lineage, the gene order is relatively stable and rearrangements, when found, are limited to tRNA genes (Grande et al., 2008; Lee et al., 2016; Uribe et al., 2016). In addition, the *trnK‐trnA‐trnR‐trnI‐trnN* cluster is usually conserved across multiple groups of mollusks (Irisarri et al., 2014; Lee et al., 2016; Osca et al., 2015); however, this cluster is broken in the mitogenome of *Cochlespira* sp., which better aligns with the hypothesis that Cochlespiridae is a sister group to all the other Conoidea rather than a sister group to Marshallenidae (Abdelkrim et al., 2018).

### 3.6 Adaptive molecular evolution

Previous studies have shown that purifying selection is the predominant force in the evolution of mitogenomes (Shen, et al., 2017; Shen, Wei, et al., 2017; Tomasco & Lessa, 2011). However, considering that mitochondria are the main location of aerobic respiration and energy metabolism, weak and/or episodic positive selection may occur against this background of strong purifying selection in locations with greater energy requirements or changing oxygen supplies. Studies have found evidence that the mitochondrial PCGs underwent positive selection in insects with flight ability, whereas no significant sign of selection was found in flightless insects where the wings had degenerated (Yang et al., 2014). Similarly, adaptive residues were also identified in mitogenomes of Ordovician bivalves before the staggering increase in atmospheric oxygen in the Lower Devonian (Plazzi et al., 2017). Here, because the deep‐sea environment may impact the functions of mitogenomes, we examined the potential positive selection pressure in the deep‐sea Conoidea lineage. Consistent results showing positive selection were obtained (Table 2), although different phylogenetic tree topologies were used.

**TABLE 2:** CODEML analyses of selective pressure on mitochondrial genes in the deep‐sea Conoidea lineage

Trees	Models	lnL	Parameter estimates	Model compared	2∆L
Branch models					
Bayesian tree	M_0_ (one ration)	−213279.037231	ω = 0.04241		
	M_1_ (free rations)	−212710.011328		M_1_ versus M_0_	1,138.05181*
	M_2_ (two rations)	−213257.130281	ω_0_ = 0.04236 ω_1_ = 0.29583	M_2_ versus M_0_	43.81390*
ML tree	M_0_ (one ration)	−213279.037231	ω = 0.04241		
	M_1_ (free rations)	−212821.045021		M_1_ versus M_0_	915.98442*
	M_2_ (two rations)	−213257.130281	ω_0_ = 0.04236 ω_1_ = 0.29583	M_2_ versus M_0_	43.81390*
Branch‐site models					
Bayesian tree	Null model	−209692.340126	P_0_ = 0.00000 P_1_ = 0.00000 P_2a_ = 0.62181 P_2b_ = 0.20137		
			ω_0_ = 0.03123 ω_1_ = 1.00000 ω_2a_ = 1.00000 ω_2b_ = 1.00000		
	Model A	−209637.335416	P_0_ = 0.61327 P_1_ = 0.08911 P_2a_ = 0.06321 P_2b_ = 0.00743	Model A versus null model	110.00942*
			ω_0_ = 0.03182 ω_1_ = 1.00000 ω_2a_ = 5.14627 ω_2b_ = 5.14627		
ML tree	Null model	−209692.340126	P_0_ = 0.00000 P_1_ = 0.00000 P_2a_ = 0.62181 P_2b_ = 0.20137		
			ω_0_ = 0.03123 ω_1_ = 1.00000 ω_2a_ = 1.00000 ω_2b_ = 1.00000		
	Model A	−209637.335416	P_0_ = 0.61104 P_1_ = 0.08910 P_2a_ = 0.06409 P_2b_ = 0.00721	Model A versus null model	110.00942*
			ω_0_ = 0.03091 ω_1_ = 1.00000 ω_2a_ = 5.01423 ω_2b_ = 5.01423		

* *p* <.001.

In the branch models, the ω ratio calculated in the M_0_ (one‐ratio model) was 0.04241 for the sampled gastropods, which indicated that the mitochondrial PCGs have experienced constrained selection pressure to maintain function (Das, 2006). M_1_ (free‐ratio model) fit the data significantly better than M_0_ (Table 2), which means that the mitochondrial PCGs have been under distinct selection pressure among different lineages of Gastropoda. Furthermore, M_2_ (two‐ratio model) also fit the data better than M_0_ (Table 2) when the deep‐sea Conoidea lineage was set as a foreground branch. In M_2_, the ω ratio of the deep‐sea Conoidea lineage (ω_1_ = 0.29583) was significantly higher than that of other shallow water gastropod species (ω_0_ = 0.04236). However, the ω ratio of the deep‐sea Conoidea lineage was still <1, suggesting that strong purifying selection played a central role in the evolution of mitochondria to maintain their important energy metabolism functions. The relaxation of selective constraints along the deep‐sea Conoidea lineage may represent an alternative compatible explanation.

**FIGURE 5:**
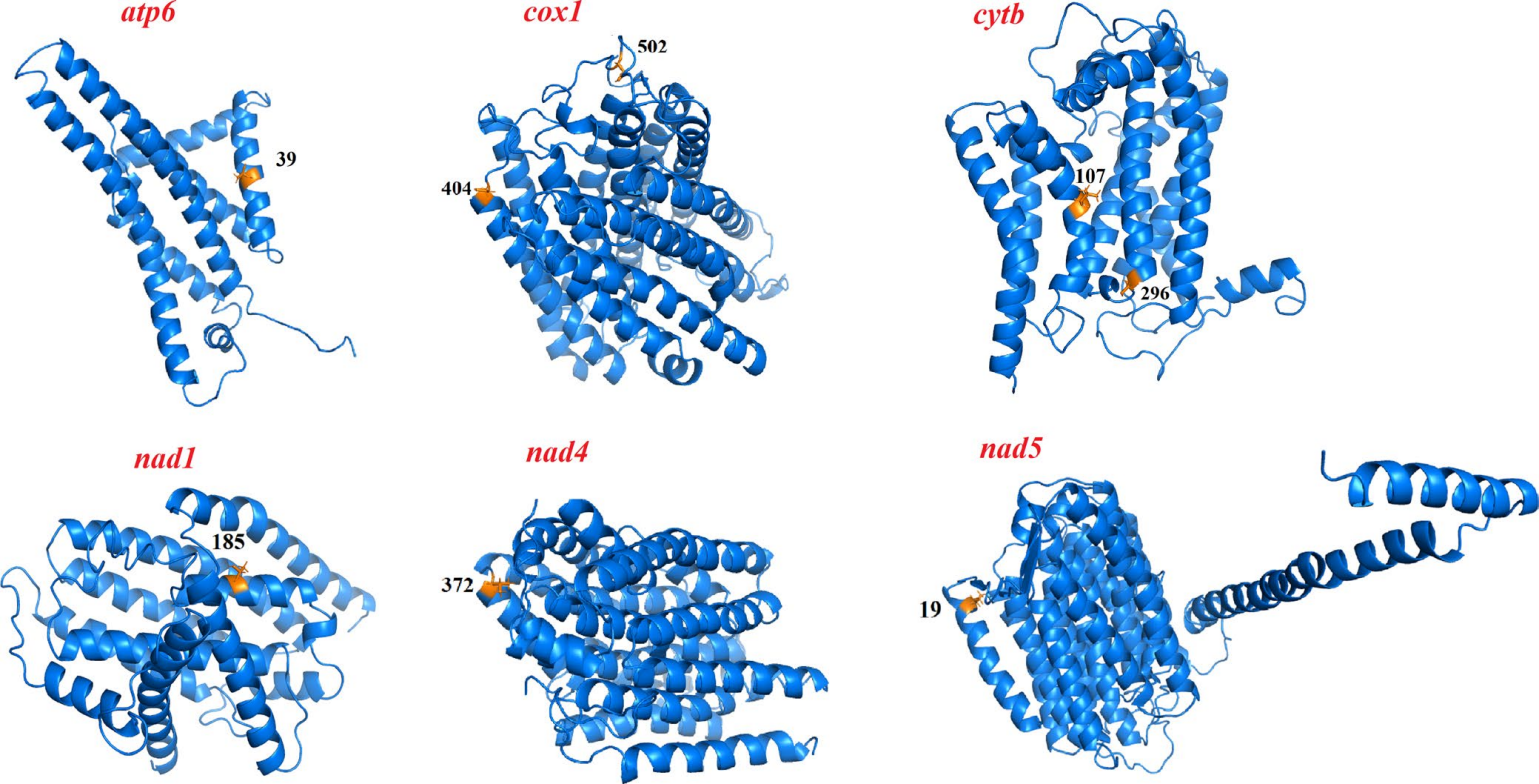
Distribution of positively selected sites in the three‐dimensional (3D) structures of atp6, cox1, cytb, nad1, nad4, and nad5

Typically, positive selection acts on only a few sites for a brief period of evolutionary history; hence, the signal for positive selection is frequently hidden in continuous purifying selection in the gene sequences (Shen, Kou, et al., 2017; Shen, Wei, et al., 2017; Zhang et al., 2005). A positively selected site is generally considered more reliable if it can be supported by two or more different methods. In the present study, branch‐site models and TreeSAAP were used to detect possible positively selected sites in the deep‐sea Conoidea lineage. Eight residues located in *atp6*, *cox1*, *cytb*, *nad1*, *nad4,* and *nad5* were determined to have undergone positive selection by both CodeML (BEB values >95%) and TreeSAAP (Table 3). The functional domains of the mitochondrial PCGs were further examined to determine the significance of the putative positively selected sites. The results showed that most positively selected sites were located in or close to the functional regions (Figure 5).

**TABLE 3:** Possible sites under positive selection of mitochondrial PCGs in the deep‐sea Conoidea lineage identified by CodeML and TreeSAAP

Bayesian tree				ML tree				TreeSAAP^a^
Gene	Positive selection sites	Amino acid	BEB values	Gene	Positive selection sites	Amino acid	BEB values	Radical changes in amino acid properties^a^
*atp6*	39	G	0.980	*atp6*	39	G	0.980	pK′
*cox1*	758	P	0.997	*cox1*	758	P	0.997	αn, Ht
	856	A	0.977		856	A	0.977	Pα
*cytb*	1,483	I	0.966	*cytb*	1,483	I	0.966	Ns
	1,672	V	0.981		1,672	V	0.981	Pβ, EI
*nad1*	1,945	V	0.982	*nad1*	1,945	V	0.982	Ns, Ra, Hp
*nad4*	3,166	S	0.992	*nad4*	3,166	S	0.992	Ns, Bl, EI, Ra, Hp
*nad5*	3,271	Y	0.955	*nad5*	3,271	Y	0.955	Ns, RF, Hp

^a^ Pα = alpha‐helical tendency, Ns =average number of surrounding residues, Pβ = beta‐structure tendency, Bl =bulkiness, Br =buriedness, RF =chromatographic index, Pc =coil tendency, c = composition, K0 = compressibility, pK′ = equilibrium constant (ionization COOH), Ca =helical contact area, h = hydropathy, pHi =isoelectric point, El =long‐range nonbonded energy, *F* = mean r.m.s. fluctuational displacement, Mv =molecular volume, Mw =molecular weight, Hnc =normalized consensus hydrophobicity, V0 = partial specific volume, Pr =polar requirement, p = polarity, αc = power to be at the C‐terminus, αm = power to be at the middle of the α‐helix, αn = power to be at the *N*‐terminus, μ = Refractive index, Esm =short‐ and medium‐range nonbonded energy, Ra =solvent accessible reduction ratio, Hp =surrounding hydrophobicity, Ht =thermodynamic transfer hydrophobicity, Et =total nonbonded energy, Pt =turn tendency.

Mitochondria can generate more than 95% of cellular energy through OXPHOS and are the energy metabolism centers of eukaryotic cells. The OXPHOS system consists of five multisubunit complexes (complexes I–V). Except for complex II, which is derived from nuclear DNA, all of the complexes are encoded by both nuclear and mitochondrial genes (Koopman et al., 2013). Complex I is the largest enzyme complex in the respiratory chain (da Fonseca et al., 2008) and functions as a proton pump, which oxidizes NADH to NAD^+^ and donates the released electrons to the electron carrier coenzyme Q10 (Koopman et al., 2010). In our work, three positively selected sites were located in the *nad1*, *nad4,* and *nad5* genes (Table 3). The deep‐sea environment is typically hypoxic and under constant darkness, high hydrostatic pressure, and low‐temperature conditions (Sanders & Hessler, 1969). Under these harsh conditions, organisms may require modified and adapted energy metabolism, and evidence of adaptive evolution in complex I has been reported in the mitogenomes of deep‐sea alvinocaridid shrimp, vesicomyids, sea anemones (*Bolocera* sp.), and sea cucumbers (*Benthodytes marianensis*) (Mu et al., 2018b; Sun et al., 2018; Yang et al., 2019; Zhang, Wu, et al., 2020; Zhang, Gao, et al., 2020). Complex III contains eleven subunits, one of which is encoded by the mitochondrial *cytb* gene (Koopman et al., 2013) and catalyzes reversible electron transfer from ubiquinol to cytochrome *c* coupled to proton translocation (Trumpower, 1990). Two residues in the *cytb* gene were found to be under positive selection (Table 3). Wide variation in the properties of amino acids was observed in functionally important regions of *cytb* in species with relatively more specialized metabolic requirements (da Fonseca et al., 2008; Silva et al., 2014). Complex IV consists of fourteen subunits, three of which are encoded by mitochondrial genes (*cox1*, *cox2,* and *cox3*) and catalyze electron donations to molecular oxygen to form water (Koopman et al., 2013). Complex IV seems to be more critical to energy supply than the other complexes because approximately 95% of the oxygen that organisms breathe is consumed by this complex (Fergusonmiller et al., 2012). The positive selection residues in complex IV suggested that deep‐sea conoidean gastropods may have adaptively enhanced oxygen use efficiency under hypoxic conditions while maintaining essential metabolic levels. Complex V (ATP synthase), which is encoded by the *atp6* and *atp8* genes, is the last enzyme complex in the respiratory chain, and it couples proton flow from the intermembrane space back to the matrix by producing ATP directly (Mishmar et al., 2003). We found one residue in the *atp6* gene under positive selection (Table 3), and studies have shown that variation in ATP synthase could enhance the ability to adapt to different environments (Sun et al., 2018; Wang, Shen, et al., 2019; Wang, Tang, et al., 2019; Xu et al., 2007; Zhang, Wu, et al., 2020; Zhang, Gao, et al., 2020). Given that limited molecular data are currently available for deep‐sea Conoidea and our samples are limited, we sincerely hope that more data will be available in the future to support the relevant research and provide insights on the mitogenome adaptation of conoidean gastropods in deep‐sea environments.

The deep‐sea environment is characterized by darkness, hypoxia, low temperatures, and high hydrostatic pressure. Under these extreme environmental conditions, a modified and adapted energy metabolism is required for survival. In the present study, eight residues located in the *atp6*, *cox1*, *cytb*, *nad1*, *nad4*, and *nad5* genes were inferred to be positively selected sites along the branches leading to deep‐sea conoidean gastropods, which indicates that the related genes were potentially under positive selection pressure. This study could help us to better understand the adaptation of organisms to the deep‐sea environment.

## AUTHOR CONTRIBUTION

Mei Yang: Conceptualization (lead); Writing‐original draft (lead). Dong Dong: Conceptualization (supporting); Writing‐review & editing (supporting). Xinzheng Li: Conceptualization (supporting); Writing‐original draft (supporting).

## Supporting information

Fig S1

Table S1

Table S2

Table S3

Table S4

## Data Availability

DNA sequences: GenBank accession number MN840973 for *Phymorhynchus* sp.
