# Supplementary figures and images for: The complete mitogenome of Phymorhynchus sp. (Neogastropoda, Conoidea, Raphitomidae) provides insights into the deep‐sea adaptive evolution of Conoidea
Source: Ecol Evol. 2021 May 2;11(12):7518–31. doi: 10.1002/ece3.7582 (PMC8216942; doi:10.1002/ece3.7582)

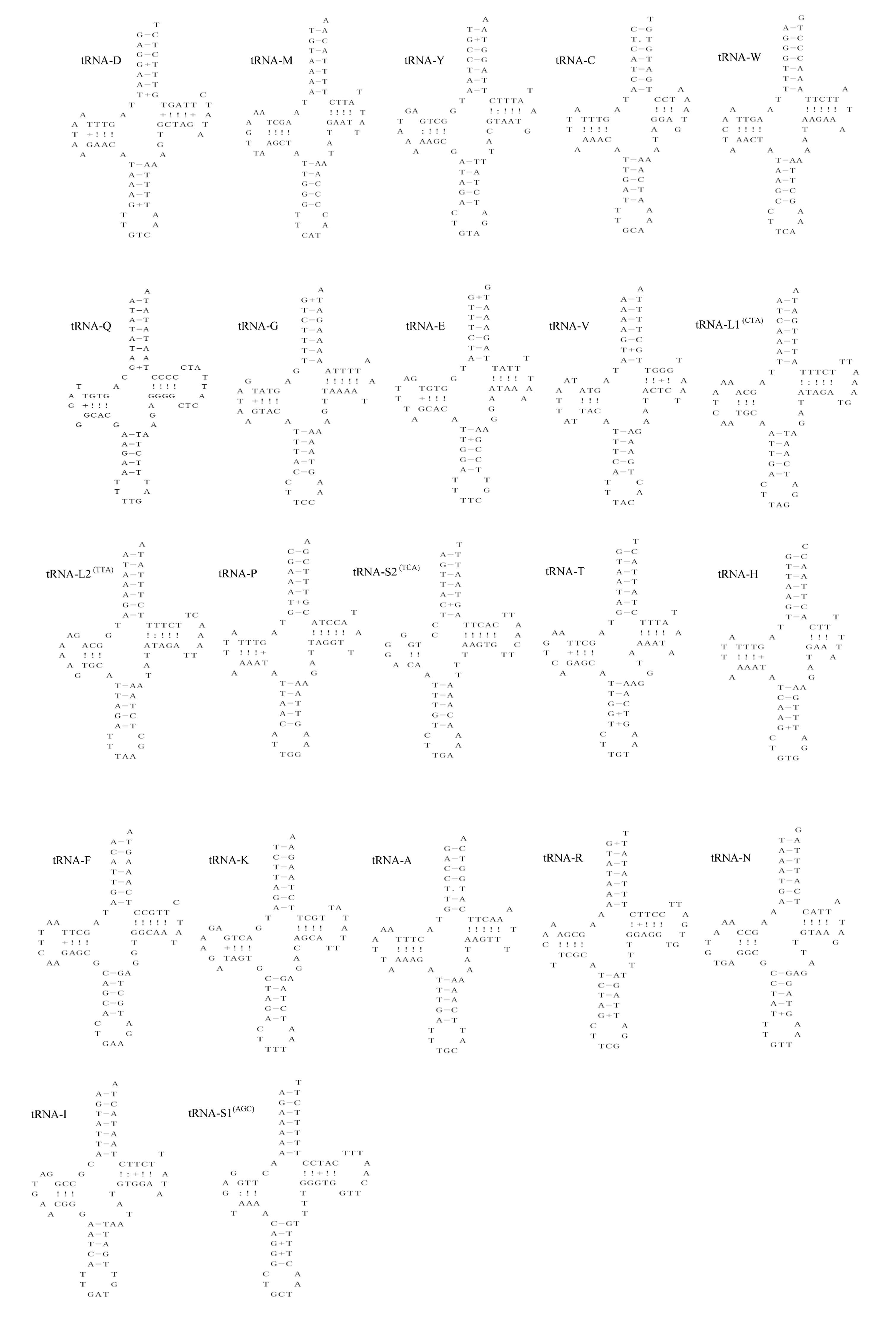

Supplement: Supplementary file 1 — Fig S1 [file ECE3-11-7518-s004.tif]
