# Supplementary material for: The complete mitogenome of Phymorhynchus sp. (Neogastropoda, Conoidea, Raphitomidae) provides insights into the deep‐sea adaptive evolution of Conoidea
Source: Ecol Evol. 2021 May 2;11(12):7518–31. doi: 10.1002/ece3.7582 (PMC8216942; doi:10.1002/ece3.7582)
Supplement: Supplementary file 2 — Table S1 [file ECE3-11-7518-s001.docx]

**TABLE S1** Species used for phylogenetic reconstructions.

| Species | Family | Genus | Accession  number | References |
| --- | --- | --- | --- | --- |
| *Bathytoma punicea* | Borsoniidae | *Bathytoma* | NC_038182 | Uribe, Zardoya, & Puillandre (2018) |
| *Tomopleura* sp. | Borsoniidae | *Tomopleura* | KX263259 | Uribe, Puillandre, & Zardoya (2017) |
| *Glyphostoma* sp. | Clathurellidae | *Glyphostoma* | KX263260 | Uribe, Puillandre, & Zardoya (2017) |
| *Clavatula tripartita* | Clavatulidae | *Clavatula* | MH308391 | Uribe, Zardoya, & Puillandre (2018) |
| *Clionella kraussii* | Clavatulidae | *Clionella* | MH308390 | Uribe, Zardoya, & Puillandre (2018) |
| *Cochlespira* sp. | Cochlespiridae | *Cochlespira* | MH308394 | Uribe, Zardoya, & Puillandre (2018) |
| *Californiconus californicus* | Conidae | *Californiconus* | NC_032377 | Uribe, Puillandre, & Zardoya (2017) |
| *Conasprella wakayamaensis* | Conidae | *Conasprella* | KX263254 | Uribe, Puillandre, & Zardoya (2017) |
| *Conus capitaneus* | Conidae | *Conus* | NC_030354 | Chen et al. (2016) |
| *Conus gloriamaris* | Conidae | *Conus* | NC_030213 | Chen et al. (Unpublished) |
| *Conus quercinus* | Conidae | *Conus* | MH400188 | Chen, Wu, & Hwang (2018) |
| *Conus striatus* | Conidae | *Conus* | KX156937 | Chen et al. (Unpublished) |
| *Conus tulipa* | Conidae | *Conus* | KR006970 | Chen et al. (2015) |
| *Lilliconus sagei* | Conidae | *Lilliconus* | KX263255 | Uribe, Puillandre, & Zardoya (2017) |
| *Pygmaeconus traillii* | Conidae | *Pygmaeconus* | KX263257 | Uribe, Puillandre, & Zardoya (2017) |
| *Splendrillia* sp. | Drilliidae | *Splendrillia* | NC_038184 | Uribe, Zardoya, & Puillandre (2018) |
| *Fusiturris similis* | Fusiturridae | *Fusiturris* | EU827197 | Cunha, Grande, & Zardoya (2009) |
| *Anguloclavus* sp. | Horaiclavidae | *Anguloclavus* | MH308399 | Uribe, Zardoya, & Puillandre (2018) |
| *Benthomangelia* sp. | Mangeliidae | *Benthomangelia* | MH308400 | Uribe, Zardoya, & Puillandre (2018) |
| *Toxicochlespira* sp. | Mangeliidae | *Toxicochlespira* | MH308401 | Uribe, Zardoya, & Puillandre (2018) |
| *Marshallena* sp. | Marshallenidae | *Marshallena* | MH308398 | Uribe, Zardoya, & Puillandre (2018) |
| *Inquisitor* sp. | Pseudomelatomidae | *Inquisitor* | MH308403 | Uribe, Zardoya, & Puillandre (2018) |
| *Leucosyrinx* sp. | Pseudomelatomidae | *Leucosyrinx* | NC_038185 | Uribe, Zardoya, & Puillandre (2018) |
| *Otitoma* sp. | Pseudomelatomidae | *Otitoma* | MH308405 | Uribe, Zardoya, & Puillandre (2018) |
| *Eubela* sp. | Raphitomidae | *Eubela* | MH308406 | Uribe, Zardoya, & Puillandre (2018) |
| *Phymorhynchus* sp. | Raphitomidae | *Phymorhynchus* | MN840973 | This study |
| *Typhlosyrinx* sp. | Raphitomidae | *Typhlosyrinx* | NC_038186 | Uribe, Zardoya, & Puillandre (2018) |
| *Oxymeris dimidiata* | Terebridae | *Oxymeris* | NC_013239 | Cunha, Grande, & Zardoya (2009) |
| *Gemmuloborsonia moosai* | Turridae | *Gemmuloborsonia* | NC_038183 | Uribe, Zardoya, & Puillandre (2018) |
| *Iotyrris cerithiformis* | Turridae | *Lophiotoma* | DQ284754 | Bandyopadhyay et al. (2006) |
| *Lucerapex* sp. | Turridae | *Lucerapex* | MH308393 | Uribe, Zardoya, & Puillandre (2018) |
| *Pinguigemmula* sp. | Turridae | *Pinguigemmula* | MH308408 | Uribe, Zardoya, & Puillandre (2018) |
| Outgroups |  |  |  |  |
| *Bolinus Brandaris* | Muricidae | *Bolinus* | NC_0113250 | Cunha, Grande, & Zardoya (2009) |
| *Nassarius javanus* | Nassariidae | *Nassarius* | NC_041547 | Yang, Li, Kong, & Yu (2019) |
| *Tritia reticulata* | Nassariidae | *Tritia* | NC_013248 | Cunha, Grande, & Zardoya (2009) |
