# Supplementary material for: The complete mitogenome of Phymorhynchus sp. (Neogastropoda, Conoidea, Raphitomidae) provides insights into the deep‐sea adaptive evolution of Conoidea
Source: Ecol Evol. 2021 May 2;11(12):7518–31. doi: 10.1002/ece3.7582 (PMC8216942; doi:10.1002/ece3.7582)
Supplement: Supplementary file 3 — Table S2 [file ECE3-11-7518-s005.docx]

**TABLE S2** Best partitioning scheme and substitution models selected by PartitionFinder 2 in this study.

| Subset | Subset Partitions | Partition delineation | Best Model |  |
| --- | --- | --- | --- | --- |
| Partition 1 | atp6_codon1, nad5_codon1 | 1-690\3, 8944-10650\3 | GTR+I+G |  |
| Partition 2 | atp6_codon2, nad1_codon2, nad3_codon2, nad4L_codon2 | 2-690\3, 4976-5913\3, 6944-7293\3, 7295-7587\3 | GTR+I+G |  |
| Partition 3 | atp6_codon3 | 3-690\3 | HKY+I+G |  |
| Partition 4 | atp8_codon1, nad2_codon1, nad6_codon1 | 691-846\3, 5914-6942\3, 10651-11145\3 | GTR+I+G |  |
| Partition 5 | atp8_codon2 | 692-846\3 | K80+I+G |  |
| Partition 6 | atp8_codon3, cox1_codon3, cox2_codon3 | 693-846\3, 849-2379\3, 2382-3060\3 | HKY+I+G |  |
| Partition 7 | cox1_codon1 | 847-2379\3 | GTR+I+G |  |
| Partition 8 | cox1_codon2 | 848-2379\3 | GTR+I+G |  |
| Partition 9 | cox2_codon1 | 2380-3060\3 | GTR+I+G |  |
| Partition 10 | cox2_codon2 | 2381-3060\3 | GTR+I+G |  |
| Partition 11 | cox3_codon1, cytb_codon1 | 3061-3837\3, 3838-4974\3 | GTR+I+G |  |
| Partition 12 | cox3_codon2, cytb_codon2 | 3062-3837\3, 3839-4974\3 | GTR+I+G |  |
| Partition 13 | cox3_codon3, nad3_codon3 | 3063-3837\3, 6945-7293\3 | HKY+I+G |  |
| Partition 14 | cytb_codon3, nad1_codon3 | 3840-4974\3, 4977-5913\3 | HKY+I+G |  |
| Partition 15 | nad1_codon1, nad3_codon1 | 4975-5913\3, 6943-7293\3 | GTR+I+G |  |
| Partition 16 | nad2_codon2, nad6_codon2 | 5915-6942\3, 10652-11145\3 | GTR+I+G |  |
| Partition 17 | nad2_codon3 | 5916-6942\3 | GTR+I+G |  |
| Partition 18 | nad4L_codon1 | 7294-7587\3 | GTR+I+G |  |
| Partition 19 | nad4L_codon3 | 7296-7587\3 | HKY+I+G |  |
| Partition 20 | nad4_codon1 | 7588-8943\3 | GTR+I+G |  |
| Partition 21 | nad4_codon2, nad5_codon2 | 7589-8943\3, 8945-10650\3 | GTR+I+G |  |
| Partition 22 | nad4_codon3 | 7590-8943\3 | GTR+I+G |  |
| Partition 23 | nad5_codon3 | 8946-10650\3 | GTR+G |  |
| Partition 24 | nad6_codon3 | 10653-11145\3 | GTR+I+G |  |
