# Supplementary material for: The complete mitogenome of Phymorhynchus sp. (Neogastropoda, Conoidea, Raphitomidae) provides insights into the deep‐sea adaptive evolution of Conoidea
Source: Ecol Evol. 2021 May 2;11(12):7518–31. doi: 10.1002/ece3.7582 (PMC8216942; doi:10.1002/ece3.7582)
Supplement: Supplementary file 4 — Table S3 [file ECE3-11-7518-s002.docx]

**Table S3.** Species used for CodeML analyses of selective pressure on mitochondrial genes.

| Species | Subclass | Order | Superfamily | Family | Accession number | Sampling depths | References |
| --- | --- | --- | --- | --- | --- | --- | --- |
| *Clavatula tripartita* | Caenogastropoda | Neogastropoda | Conoidea | Clavatulidae | MH308391 | 52 m | Uribe, Zardoya, & Puillandre (2018) |
| *Clionella kraussii* | Caenogastropoda | Neogastropoda | Conoidea | Clavatulidae | MH308390 | 20-22 m | Uribe, Zardoya, & Puillandre (2018) |
| *Conasprella wakayamaensis* | Caenogastropoda | Neogastropoda | Conoidea | Conidae | KX263254 | 335 m | Uribe, Puillandre, & Zardoya (2017) |
| *Eubela sp.* | Caenogastropoda | Neogastropoda | Conoidea | Raphitomidae | MH308406 | >600 m | Uribe, Zardoya, & Puillandre (2018) |
| *Euspira gilva* | Caenogastropoda | Littorinimorpha | Naticoidea | Naticidae | MK478016 | <200 m | Liu et al. (2020) |
| *Fusinus longicaudus* | Caenogastropoda | Neogastropoda | Buccinoidea | Fasciolariidae | NC_045906 | <200 m | Feng et al. (2019) |
| *Fusiturris similis* | Caenogastropoda | Neogastropoda | Conoidea | Fusiturridae | EU827197 | <200 m | Cunha, Grande, & Zardoya (2009) |
| *Inquisitor sp.* | Caenogastropoda | Neogastropoda | Conoidea | Pseudomelatomidae | MH308403 | >600 m | Uribe, Zardoya, & Puillandre (2018) |
| *Leucosyrinx sp.* | Caenogastropoda | Neogastropoda | Conoidea | Pseudomelatomidae | NC_038185 | >500 m | Uribe, Zardoya, & Puillandre (2018) |
| *Littorina brevicula* | Caenogastropoda | Littorinimorpha | Littorinoidea | Littorinidae | NC_050987 | <200 m | Bai et al. (2020) |
| *Mitromorpha fischeri* | Caenogastropoda | Neogastropoda | Conoidea | Mitromorphidae | MH308402 | 12 m | Uribe, Zardoya, & Puillandre (2018) |
| *Nassarius glans* | Caenogastropoda | Neogastropoda | Buccinoidea | Nassariidae | NC_049091 | <200 m | Yang et al. (2020) |
| *Nassarius javanus* | Caenogastropoda | Neogastropoda | Buccinoidea | Nassariidae | NC_041547 | <200 m | Yang, Li, Kong, & Yu (2019) |
| *Nassarius siquijorensis* | Caenogastropoda | Neogastropoda | Buccinoidea | Nassariidae | NC_048962 | <200 m | Yang et al. (2020) |
| *Neverita didyma* | Caenogastropoda | Littorinimorpha | Naticoidea | Naticidae | MK478017 | <200 m | Liu et al. (2020) |
| *Oxymeris dimidiata* | Caenogastropoda | Neogastropoda | Conoidea | Terebridae | NC_013239 | <200 m | Cunha, Grande, & Zardoya (2009) |
| *Profundiconus teramachii* | Caenogastropoda | Neogastropoda | Conoidea | Conidae | KX263254 | 540-580 m | Uribe, Puillandre, & Zardoya (2017) |
| *Phymorhynchus sp.* | Caenogastropoda | Neogastropoda | Conoidea | Raphitomidae | MN840973 | >1000 m | This study |
| *Toxicochlespira sp.* | Caenogastropoda | Neogastropoda | Conoidea | Mangeliidae | MH308401 | >600 m | Uribe, Zardoya, & Puillandre (2018) |
| *Typhlosyrinx sp.* | Caenogastropoda | Neogastropoda | Conoidea | Raphitomidae | NC_038186 | >600 m | Uribe, Zardoya, & Puillandre (2018) |
