# Supplementary material for: The complete mitogenome of Phymorhynchus sp. (Neogastropoda, Conoidea, Raphitomidae) provides insights into the deep‐sea adaptive evolution of Conoidea
Source: Ecol Evol. 2021 May 2;11(12):7518–31. doi: 10.1002/ece3.7582 (PMC8216942; doi:10.1002/ece3.7582)
Supplement: Supplementary file 5 — Table S4 [file ECE3-11-7518-s003.docx]

**TABLE S4** Genomic features of the mitogenomes of Conoidea species.

| Species | Family | Genus | Accession  number | Length  (bp) | Genome | | | Protein-coding gene | | | rRNAs | | tRNAs | |
| --- | --- | --- | --- | --- | --- | --- | --- | --- | --- | --- | --- | --- | --- | --- |
|  |  |  |  |  | AT% | AT  skew | GC  skew | Length  (aa) | AT%  (all) | AT%  (3rd) | Length  (bp) | AT% | Number/  Length(bp) | AT% |
| *Bathytoma punicea* | Borsoniidae | *Bathytoma* | NC_038182 | 16037 | 66.0 | 0.115 | -0.004 | 3738 | 64.8 | 73.2 | 2298 | 70.3 | 22/1470 | 67.1 |
| *Tomopleura* sp. * | Borsoniidae | *Tomopleura* | KX263259 | 15182 | 69.3 | 0.192 | -0.192 | 3729 | 68.1 | 79.6 | 2309 | 73.2 | 21/1425 | 71.6 |
| *Glyphostoma* sp. * | Clathurellidae | *Glyphostoma* | KX263260 | 13376 | 68.4 | 0.166 | -0.161 | 3117 | 67.1 | 79.5 | 2323 | 70.8 | 20/1350 | 69.0 |
| *Clavatula tripartita** | Clavatulidae | *Clavatula* | MH308391 | 15743 | 68.5 | 0.113 | -0.027 | 3731 | 67.1 | 79.0 | 2327 | 70.6 | 21/1433 | 67.8 |
| *Clionella kraussii** | Clavatulidae | *Clionella* | MH308390 | 15760 | 68.8 | 0.105 | -0.009 | 3717 | 67.3 | 78.9 | 2341 | 71.3 | 21/1415 | 66.3 |
| *Cochlespira* sp. * | Cochlespiridae | *Cochlespira* | MH308394 | 15581 | 63.9 | 0.024 | 0.173 | 3721 | 61.9 | 67.6 | 2303 | 69.6 | 21/1416 | 65.9 |
| *Californiconus californicus* | Conidae | *Californiconus* | NC_032377 | 15444 | 65.8 | 0.131 | -0.020 | 3737 | 64.5 | 74.5 | 2312 | 69.1 | 22/1485 | 66.6 |
| *Conasprella wakayamaensis** | Conidae | *Conasprella* | KX263254 | 15927 | 69.4 | 0.131 | -0.041 | 3739 | 68.1 | 82.3 | 2334 | 71.3 | 21/1438 | 68.6 |
| *Conus gloriamaris* | Conidae | *Conus* | NC_030213 | 15774 | 66.2 | 0.163 | -0.108 | 3741 | 65.9 | 76.8 | 2333 | 68.4 | 22/1488 | 64.4 |
| *Conus quercinus* | Conidae | *Conus* | MH400188 | 16439 | 66.3 | 0.153 | -0.116 | 3742 | 65.8 | 76.1 | 2328 | 67.5 | 22/1482 | 65.7 |
| *Conus striatus* | Conidae | *Conus* | KX156937 | 15738 | 64.5 | 0.196 | -0.174 | 3741 | 64.1 | 71.9 | 2317 | 66.0 | 22/1477 | 62.7 |
| *Conus tulipa* | Conidae | *Conus* | KR006970 | 15756 | 66.4 | 0.140 | -0.100 | 3742 | 65.9 | 76.9 | 2322 | 67.9 | 22/1486 | 64.3 |
| *Lilliconus sagei** | Conidae | *Lilliconus* | KX263255 | 15665 | 72.9 | 0.130 | -0.038 | 3735 | 71.7 | 87.6 | 2381 | 74.9 | 20/1362 | 74.4 |
| *Profundiconus teramachii** | Conidae | *Profundiconus* | KX263256 | 15279 | 69.1 | 0.123 | -0.025 | 3734 | 68.3 | 82.5 | 2343 | 71.1 | 21/1435 | 69.5 |
| *Pygmaeconus traillii** | Conidae | *Pygmaeconus* | KX263257 | 14963 | 70.2 | 0.120 | -0.025 | 3737 | 69.2 | 80.8 | 2236 | 73.3 | 21/1398 | 70.7 |
| *Splendrillia* sp. | Drilliidae | *Splendrillia* | NC_038184 | 15358 | 70.9 | 0.111 | -0.036 | 3733 | 70.1 | 84.9 | 2342 | 73.9 | 22/1478 | 68.8 |
| *Anguloclavus* sp. * | Horaiclavidae | *Anguloclavus* | MH308399 | 15103 | 67.0 | 0.048 | 0.123 | 3722 | 65.8 | 77.0 | 2310 | 71.3 | 21/1399 | 66.2 |
| *Benthomangelia* sp.* | Mangeliidae | *Benthomangelia* | MH308400 | 15037 | 71.5 | 0.115 | -0.032 | 3735 | 70.8 | 86.4 | 2285 | 74.5 | 21/1384 | 69.2 |
| *Toxicochlespira* sp. * | Mangeliidae | *Toxicochlespira* | MH308401 | 15076 | 72.1 | 0.112 | -0.036 | 3733 | 71.7 | 88.3 | 2287 | 74.1 | 21/1388 | 69.7 |
| *Marshallena* sp. * | Marshallenidae | *Marshallena* | MH308398 | 15210 | 65.9 | 0.066 | 0.082 | 3731 | 64.2 | 71.7 | 2317 | 71.0 | 21/1414 | 67.0 |
| *Mitromorpha fischeri** | Mitromorphidae | *Mitromorpha* | MH308402 | 15243 | 67.0 | 0.122 | -0.045 | 3735 | 66.1 | 76.5 | 2315 | 68.7 | 21/1402 | 68.3 |
| *Eubela* sp.* | Raphitomidae | *Eubela* | MH308406 | 15153 | 69.9 | 0.113 | -0.037 | 3733 | 68.4 | 81.0 | 2255 | 74.4 | 21/1382 | 70.0 |
| *Phymorhynchus* sp. | Raphitomidae | *Phymorhynchus* | MN840973 | 16681 | 69.5 | -0.102 | 0.034 | 3702 | 67.9 | 81.3 | 2235 | 73.0 | 22/1444 | 70.4 |
| *Typhlosyrinx* sp. | Raphitomidae | *Typhlosyrinx* | NC_038186 | 15804 | 70.5 | 0.112 | -0.044 | 3740 | 69.0 | 81.5 | 2340 | 74.1 | 22/1440 | 71.2 |
| *Oxymeris dimidiata* | Terebridae | *Oxymeris* | NC_013239 | 16513 | 65.6 | 0.219 | -0.265 | 3732 | 65.0 | 71.6 | 2352 | 65.9 | 22/1491 | 65.8 |
| *Fusiturris similis* | Fusiturridae | *Fusiturris* | EU827197 | 15595 | 66.4 | 0.124 | -0.043 | 3735 | 64.9 | 73.7 | 2309 | 71.0 | 22/1485 | 68.1 |
| *Gemmuloborsonia moosai* | Turridae | *Gemmuloborsonia* | NC_038183 | 15541 | 68.2 | 0.119 | -0.021 | 3730 | 67.1 | 79.2 | 2318 | 72.3 | 22/1489 | 66.9 |
| *Iotyrris cerithiformis* | Turridae | *Lophiotoma* | NC_008098 | 15380 | 67.8 | 0.121 | -0.030 | 3726 | 66.7 | 77.1 | 2338 | 71.8 | 22/1494 | 68.9 |
| *Lucerapex* sp. * | Turridae | *Lucerapex* | MH308393 | 15338 | 67.9 | 0.122 | -0.023 | 3724 | 66.2 | 76.6 | 2305 | 73.2 | 21/1415 | 68.0 |
| *Pinguigemmula* sp. * | Turridae | *Pinguigemmula* | MH308408 | 15097 | 69.8 | 0.124 | -0.050 | 3719 | 68.9 | 83.1 | 2319 | 73.0 | 21/1418 | 69.5 |
| *Inquisitor* sp. * | Pseudomelatomidae | *Inquisitor* | MH308403 | 15248 | 67.4 | 0.116 | -0.031 | 3730 | 66.5 | 77.8 | 2310 | 70.8 | 22/1480 | 66.5 |
| *Leucosyrinx* sp. | Pseudomelatomidae | *Leucosyrinx* | NC_038185 | 15358 | 69.0 | 0.121 | -0.023 | 3731 | 68.3 | 82.0 | 2340 | 71.9 | 22/1477 | 68.2 |
| *Otitoma* sp. * | Pseudomelatomidae | *Otitoma* | MH308405 | 15,584 | 70.1 | 0.117 | -0.038 | 3731 | 69.5 | 84.5 | 2327 | 73.4 | 22/1493 | 68.7 |

* Incomplete mitogenome.

References

Bai, J., Guo, Y., Feng, J., Ye, Y., Li, J., Yan, C., & Mao, S. (2020). The complete mitochondrial genome and phylogenetic analysis of Littorina brevicula (Gastropoda, Littorinidea). Mitochondrial DNA B Resource, 5, 2280–2281.

Bandyopadhyay, P. K., Stevenson, B. J., Cady, M. T., Olivera, B. M., & Wolstenholme, D. R. (2006). Complete mitochondrial DNA sequence of a Conoidean gastropod, Lophiotoma (Xenuroturris) cerithiformis: Gene order and gastropod phylogeny. Toxicon, 48, 29–43. https://doi.org/10.1016/j.toxicon.2006.04.013

Chen, P. W., Hsiao, S. T., Chen, K. S., Tseng, C. T., Wu, W. L., & Hwang, D. F. (2016). The complete mitochondrial genome of Conus capitaneus (Neogastropoda: Conidae). Mitochondrial DNA Part B, 1, 520–521. https://doi.org/10.1080/23802359.2016.1197060

Chen, P. W., Hsiao, S. T., Huang, C. W., Chen, K. S., Tseng, C. T., Wu, W. L., & Hwang, D. F. (2015). The complete mitochondrial genome of Conus tulipa (Neogastropoda: Conidae). Mitochondrial DNA Part A, 27, 2738–2739. https://doi.org/10.3109/19401736.2015.1046172

Chen, P. W., Wu, W. L., & Hwang, D. F. (2018). The complete mitochondrial genome of Conus quercinus (Neogastropoda: Conidae). Mitochondrial DNA Part B, 3, 933–934. https://doi.org/10.1080/23802359.2018.1501314

Feng, J., Fu, Z., Guo, Y., Ye, Y., Li, J., Guo, B., & Lue, Z. (2019). The complete mitochondrial genome and phylogenetic analysis of Fusinus longicaudus (Gastropoda: Fasciolariidae). Mitochondrial DNA B Resource, 4, 1943–1944.

Liu, H., Yang, Y., Sun, S., Kong, L., & Li, Q. (2020). Mitogenomic phylogeny of the Naticidae (Gastropoda: Littorinimorpha) reveals monophyly of the Polinicinae. Zoologica Scripta, 49, 295–306. https://doi.org/10.1111/zsc.12412

Yang, Y., Li, Q., Kong, L., & Yu, H. (2019). Mitogenomic phylogeny of Nassarius (Gastropoda: Neogastropoda). Zoologica Scripta, 48, 302–312. https://doi.org/10.1111/zsc.12343

Yang, Y., Liu, H., Qi, L., Kong, L., & Li, Q. (2020). Complete mitochondrial genomes of two toxin‐accumulated nassariids (Neogastropoda: Nassariidae: Nassarius) and their implication for phylogeny. International Journal of Molecular Sciences, 21(10), 3545. https://doi.org/10.3390/ijms21103545

Zhang, B., Zhang, Y. H., Wang, X., Zhang, H. X., & Lin, Q. (2017). The mitochondrial genome of a sea anemone Bolocera sp. exhibits novel genetic structures potentially involved in adaptation to the deep‐sea environment. Ecology and Evolution, 7, 4951–4962. https://doi.org/10.1002/ece3.3067
